# A Comparison of Two-Dimensional and Three-Dimensional Techniques for Kinematic Analysis of the Sagittal Motion of Sheep Hindlimbs During Walking on a Treadmill

**DOI:** 10.3389/fvets.2021.545708

**Published:** 2021-08-17

**Authors:** Camila Cardoso Diogo, José Arthur Camassa, Bárbara Fonseca, Luís Maltez da Costa, José Eduardo Pereira, Vítor Filipe, Pedro Alexandre Couto, Stefania Raimondo, Paulo A. Armada-da-Silva, Ana Colette Maurício, Artur S. P. Varejão

**Affiliations:** ^1^Department of Veterinary Sciences, University of Trás-os-Montes e Alto Douro, Vila Real, Portugal; ^2^Animal and Veterinary Research Center (CECAV), Centre for Animal Sciences and Veterinary Studies, University of Trás-os-Montes e Alto Douro, Vila Real, Portugal; ^3^Department of Engineering, School of Science and Technology, University of Trás-os-Montes e Alto Douro, Vila Real, Portugal; ^4^Instituto de Engenharia de Sistemas e Computadores, Tecnologia e Ciência (INESC TEC), Rua Dr. Roberto Frias, Porto, Portugal; ^5^Centre for the Research and Technology of Agro-Environmental and Biological Sciences (CITAB), University of Trás-os-Montes e Alto Douro, Vila Real, Portugal; ^6^Department of Clinical and Biological Sciences, Neuroscience Institute Cavalieri Ottolenghi, University of Torino, Turin, Italy; ^7^Faculdade de Motricidade Humana, Universidade de Lisboa, Dafundo, Portugal; ^8^Centro Interdisciplinar de Estudo de Performance Humana, Faculdade de Motricidade Humana, Universidade de Lisboa, Dafundo, Portugal; ^9^Department of Veterinary Clinics, Institute of Biomedical Sciences Abel Salazar (ICBAS), University of Porto, Porto, Portugal; ^10^Animal Science and Study Centre (CECA), Instituto de Ciências, Tecnologias e Agroambiente da Universidade do Porto (ICETA), Porto, Portugal

**Keywords:** sheep, 2D and 3D gait analysis, kinematics, locomotion, hindlimb, treadmill

## Abstract

Compared to rodents, sheep offer several attractive features as an experimental model for testing different medical and surgical interventions related to pathological gait caused by neurological diseases and injuries. To use sheep for development of novel treatment strategies in the field of neuroscience, it is key to establish the relevant kinematic features of locomotion in this species. To use sheep for development of novel treatment strategies in the field of neuroscience, it is crucial to understand fundamental baseline characteristics of locomotion in this species. Despite their relevance for medical research, little is known about the locomotion in the ovine model, and next to nothing about the three-dimensional (3D) kinematics of the hindlimb. This study is the first to perform and compare two-dimensional (2D) and 3D hindlimb kinematics of the sagittal motion during treadmill walking in the ovine model. Our results show that the most significant differences took place throughout the swing phase of the gait cycle were for the distal joints, ankle and metatarsophalangeal joint, whereas the hip and knee joints were much less affected. The results provide evidence of the inadequacy of a 2D approach to the computation of joint kinematics in clinically normal sheep during treadmill walking when the interest is centered on the hoof's joints. The findings from the present investigation are likely to be useful for an accurate, quantitative and objective assessment of functionally altered gait and its underlying neuronal mechanisms and biomechanical consequences.

## Introduction

Studies on locomotor behavior in quadruped animals has been critical for understanding walking mechanics (1). There are numerous neurological diseases, which results in a wide-ranging and variable presentation of gait abnormalities. Gait analysis of animal models have been used to record joints and limb segments in multiple neuropathological conditions. Rodents have traditionally been considered the preferred model for testing different interventions, such as motor rehabilitation and regenerative treatments related to neurological diseases and injuries of the nervous system due to cost reduction and ease of genetic modification methods. For example, rodent models of degenerative diseases such as Alzheimer's disease and Parkinson's disease, cerebrovascular disease, traumatic injury of the spinal cord or peripheral nervous have been developed to test new medical and surgical treatments, and neurorehabilitation techniques for such neurological diseases and injuries (1–6).

Although rodents have been intensively used for research, the rate of successful translation from rodent models to clinical applications is low and are driving scientists to pursue different animal models. One way to mitigate the translational gap between experimental models and people is to start with large mammals, which includes species more closely related to humans. Utilization of large animal models that best mimic human diseases are required to improve treatment and prognosis of patients (7, 8). Modeling different neurological conditions in sheep offers several appealing features. First, similarities between human and sheep anatomy and physiology has converted the sheep a translatable model for investigating, for example, spinal cord and peripheral nerve injury (9). Second, they are attractive models for biomedical and basic biological research because of availability and they can be kept in natural groups with very basic housing demands. Third, they represent a less expensive alternative. Fourth, they are placid animals and can be readily trained for gait analysis. Fifth, the larger size of the sheep, with body weight similar to humans, facilitating biomechanical measurement of joint kinematics. Finally, other large mammals such as dogs and cats, are considered non-desirable models by society, due to their domestic relationship to humans (10).

Animal studies using the sheep as kinematic model are scarce in the literature and only in the field of orthopedics and spinal cord and peripheral nerve injury research (11–14). To improve our understanding of the pathological gait requires determination of the kinematics and biomechanics of locomotion in clinically normal sheep. Experiments using motorized treadmills are frequently applied in animal studies as they display unique biomechanical features when compared to overground walking (15, 16).

Both two-dimensional (2D) and three-dimensional (3D) systems have been used for quadruped gait analysis (17, 18). A considerable advantage of using a 2D kinematic approach is the simplicity of the procedure and the associated costs which require a single digital camera to capture the movement. This biomechanical analysis is only precise when the angular joints lies entirely in one plane of motion. Therefore, 2D systems suffer from parallax error, which occurs when the animal is in motion and is shot from different angles and perspective error, when there is rotation of segments out of the calibrated plane of motion (19). 3D gait analysis research has been used to accurately measure multiplanar and dimensional kinematics and is considered the “gold standard” in biomechanics. However, these motion capture systems are expensive due to the use of specialized equipment and operating staff and are not routinely available.

In the light of reported literature, no comparisons have been made between 2D and 3D hindlimb kinematics during treadmill walking in the ovine model. Understanding how the 2D and 3D motion capture analysis determine the waveform shapes of all joints is important for the researcher in order to decide which technique to apply for an accurate locomotion assessment. The objective of this investigation was to compare the kinematic output of the sagittal motion using 2D and 3D approach in clinically normal sheep during treadmill walking and create a template for an objective assessment of quantitative angular change for use in different neurological diseases and injuries.

## Materials and Methods

### Ovine Model

This investigation was approved by the Institutional Animal Care and Use Committee of the University of Trás-os-Montes e Alto Douro (IACUC Approval No. 6/2015). All procedures were performed with the approval of the Portuguese Veterinary Authorities, in accordance with the EU Directive 2010/63/EU for animal experiments. Six female Portuguese Churra-da-Terra Quente breed experimental sheep (age: ≈ 2 years; weight: ≈ 40 kg). Upon delivery, all animals were examined by a large animal veterinarian and deemed non-pregnant and free of disease. The sheep were judged to be healthy based on complete physical, orthopedic, and neurologic examinations. The animals were fed a diet of hay supplemented with concentrate according to their requirements and had free access to fresh water.

### Training Procedure

Two weeks before the collection of kinematic data, sheep were trained daily to walk consistently on a treadmill (Medium Fit Fur Life, Surrey, United Kingdom), with a walking area of 2.0 m length by 0.44 m width. Each sheep was led by the same handler, using a halter, onto the stationary treadmill belt. Sheep were allowed to become familiarized with the treadmill and given food rewards as positive reinforcement. The treadmill belt was started at the slowest speed possible and then the velocity was progressively augmented to reach a maximum of 1.2 m/s. The desired end-result was that the sheep would walk at a constant and comfortable walking speed on the treadmill. The hoof trimming was done at the beginning of the familiarization process.

### Kinematic Recording

**Figure 1:**
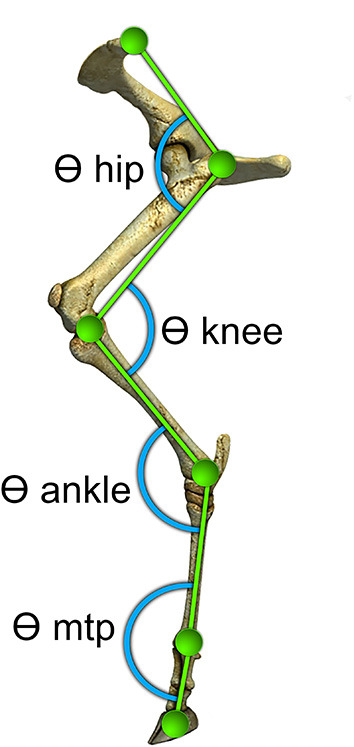
Locations of markers relative to underlying bony landmarks used to identify five segments of the sheep's left hindlimb. Hemispherical markers were attached to the skin over: the iliac crest, the greater trochanter, lateral malleolus, distal end of the metatarsal bone, and distal end of the middle phalanx. Angles (θ) at the hip, knee, ankle and metatarsophalangeal (MTP) were determined from the relative orientation of the adjacent segments and measured at the flexor side of each joint as illustrated.

To prepare the skin for easier attachment of the markers, the sheep's left hindlimbs were sheared before the recording day. Hemispherical green polystyrene markers with a diameter of 2 cm were attached to the skin by double-adhesive tape over six anatomic landmarks on the lateral side of the left hindlimb: the dorsal point of the iliac crest, the greater trochanter, the estimated joint center of the knee, the lateral malleolus, the distal end of the metatarsal bone and distal end of the middle phalanx. The same operator performed all marker placements to avoid experimental error. The hindlimb joint angles were measured at the flexor side of each joint (Figure 1).

**Figure 2:**
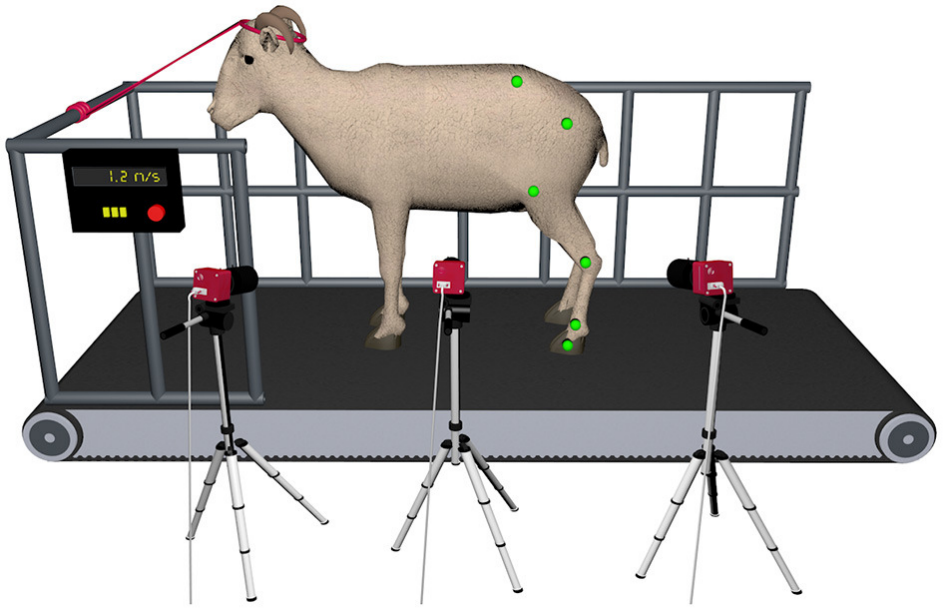
Depiction of the gait laboratory set up for simultaneous 2D and 3D hindlimb gait analysis in the ovine model during treadmill walking. Three high-speed digital cameras were strategically positioned around the left hindlimb to minimize marker occlusion and maximize resolution.

To obtain and process the kinematic data of gait, we used a 3D motion capture system developed internally in our lab. This system is composed of three synchronized CMOS cameras (PhotonFocus MV-D640C, Lachen, Switzerland) to record the videos sequences of the movement. The cameras were strategically placed around the left hindlimb to minimize marker occlusion, maximize resolution and improve the accuracy of the 3D reconstruction process. The camera in the middle, which was placed perpendicular to the direction of the movement, was used for 2D analysis. Kinematic data were collected at a sampling rate of 144 Hz. The field of view for the cameras was calibrated to cover 2 meters in length of the treadmill apparatus and was able to record 10 consecutive steps (Figure 2). The images were acquired using the software Video Savant 4 (IO Industries Inc., Ontario, Canada). The color image had a resolution of 640 × 480 pixels. After recording, the videos were offline processed by computer vision algorithms to automatically track the position of landmarks in the image plane and reconstruct the markers 3D trajectories. The Direct Linear Transformation algorithm (DLT) was used to transform the image coordinates into 3D spatial data (20).

The camera calibration and the 3D reconstruction process followed a procedure similar to the one previously detailed (21). We designed a new calibration object, which allowed us to calibrate all the three cameras at the same time. The use of the calibration object ensured that the projection matrixes were all related to the same coordinate system. This allowed all points to be used to compute the kinematic parameters without any need for their transformation. In order to measure the accuracy of the 3D reconstruction process, the known 3D coordinates of the calibration object were compared with the 3D coordinates measured and for each point a position error was calculated. This error was computed as the Euclidean distance between the measured and the reconstructed 3D coordinate. We obtained a mean absolute position error of 3.41 ± 2.9 mm.

Applying the DLT coefficients determined in the calibration stage, the three-dimensional reconstruction process receives the marker image coordinates (*x, y*) in each one of the cameras and calculates the landmark world coordinates, (*X, Y*) in the 2D analysis and (*X, Y Z*) in 3D analysis. The hindlimb segments were defined by two landmarks and the joint angles, defined by two segments with a common point, were calculated using equation 1:

(1) $$\begin{array}{l} \cos\left(\theta \right)=\frac{\vec{\text{A}}\;.\;\vec{\;\text{B}}}{\left||\text{A}||\;||\text{B}|\right|} \end{array}$$

Where $\vec{\text{A}}.\vec{\text{B}}$ represent the dot product between 2 segment vectors, ||**A**|| **and** ||**B**|| are the length of vectors, and cos(θ) is the cosine of the angle of the joint. Joint flexion-extension angles, at the flexor side for hip, knee, ankle, and metatarsophalangeal (MTP) joints were measured in the sagittal plane for comparing the 2D and 3D kinematic analysis.

A trial was considered valid if the sheep walked comfortably in a straight line without either hesitation or running. To correct for skin motion artifacts at the knee, two different methodologies were used in order to adjust the coordinates of the knee marker in the sagittal plane. For the 2D approach, the knee position was computed indirectly by superimposing two circles (centered on hip and ankle pivots) with a radius of the femur and tibia length, respectively. The knee position was determined as the intersection of the two circles. These bone lengths were obtained from radiographic images. The animals were anesthetized and radiographed in dorsal recumbency with the hindlimbs outstretched using a radiographic unit (Philips Optimus 80, Hamburg, Germany). Radiographs were taken with a setting of 60 kVp and 15 mAs. The images were obtained using Kodak film and were processed routinely using an automated radiograph developer. The measurements were made to the nearest millimeter. For the 3D biomechanical model, we applied a slightly different approach. The two circles were replaced by two spheres centered on the greater trochanter and the lateral malleolus with a radius equal to the length of the femur and tibia segments, respectively. The outer intersection points of the two spheres forms a circle.

**Figure 3:**
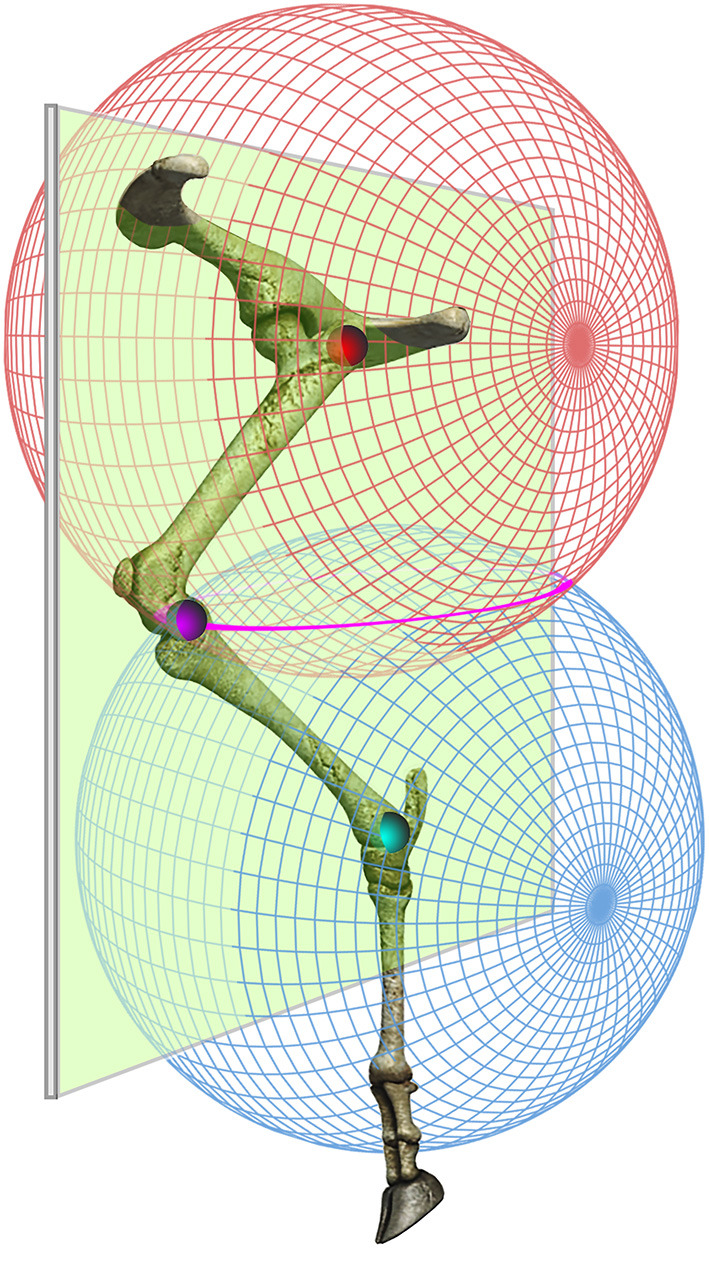
Three-dimensional estimation of the knee marker. The knee position was computed indirectly by superimposing two spheres centered on the 3D positions of the greater trochanter (red) and the lateral malleolus (blue) and with a radius equal to the length of the femur and tibia segments, respectively. It was presumed that the true position of the knee marker would lie on the plane defined by the greater trochanter, the knee and the lateral malleolus markers. From the intersection of this plane with the circle (pink) resulting from the intersection of the two spheres, we obtained two points. We chose the one that was consistent with the direction of movement (For interpretation of the references to color in this figure legend, the reader is referred to the web version of the article).

We presumed that the true position of the knee marker would lie on the plane defined by the greater trochanter, the knee and the lateral malleolus markers. From the intersection of this plane with the circle resulting from the intersection of the two spheres, two points are obtained. We chose as the knee marker the one that was consistent with the direction of movement, as previously described (Figure 3) (21).

### Numerical Analysis

The marker-based angular kinematic curves were filtered using a fourth-order Butterworth filter (cut-off frequency at 10 Hz). The gait cycle, which is the basic unit of measurement in gait analysis, was split into two parts, the stance and the swing phases. The stance phase was defined as the part of the gait cycle that begins as soon as the hoof contacts the treadmill belt and ceases when the hoof is lifted from the belt and starts its forward movement. The swing phase was considered to begin at the onset of forward movement and to terminate as the hoof strikes the treadmill belt. For each stride, the duration of the stance and swing phases was normalized. Cubic spline interpolation was applied to the original data regarding the angular position of the pelvis, hip, knee, ankle, and MTP joints to obtain 101 samples per gait cycle regardless of their duration. Maximal and minimal joint flexion-extension angles were recorded during both the stance and swing phases of gait as well as the angles at the points of initial hoof treadmill contact (initial contact, IC) (start of the stance phase) and immediately when the hoof is lifted from the treadmill (toe off, TO) (start of the swing phase). This numerical treatment was performed with Matlab computational software (The MathWorks Inc., Natick, MA, USA).

In addition, the following spatio-temporal data were included: gait cycle duration, stance duration, swing duration and stride length. The stride length of the hindlimbs was established by the distance between the middle phalanx markers in two consecutive steps.

### Statistical Analysis

Differences in joint kinematics data collected in 2D and 3D for each tested hindlimb joint and defined time point were tested by paired Student's *t*-test. Mean ± standard deviation (S.D.) values for all the measured variables are reported. Preliminary analysis of intra-trial, inter-step reliability of MTP, ankle, knee, and hip angle measurements was performed employing the two-way mixed model intra-class correlation coefficient (ICC) for absolute agreement. The differences between 2D and 3D values were deemed significant at uncorrected *p* < 0.05. All statistical tests were performed using IBM SPSS Statistics V22.0 software.

To ascertain the robustness of the pairwise comparisons, it was performed a bootstrapping procedure for each of the joint angle parameters displaying significant differences between 2D and 3D analysis. For bootstrapping, 500 samples were drawn from the original 2D and 3D data using replacement and next used to perform an equal number of separate pairwise t-tests. The bootstrapping *t*-test procedure gave rise to a set of *p*-values with a given distribution, from which the percentage of tests having *p*-values equal or above 0.05 could be derived.

## Results

To ensure a reliable comparison between the 2D and 3D sagittal plane kinematics of the hindlimb joints, a total of 60 gait cycles were obtained during treadmill locomotion from 6 clinically healthy sheep. Ten consecutive steps were analyzed for each sheep without stopping, hesitating, or running.

Healthy sheep walked in the treadmill with a speed of 1.2 m/s, a gait cycle duration of 744 ± 45 ms, a stance duration of 442 ± 31 ms, a swing duration of 302 ± 30 ms and a stride length of 83.3 ± 5.6 cm.

### Angular Kinematic Parameters

Figure 3 shows the average 2D and 3D joint kinematics of the hip, knee, ankle and metatarsophalangeal joint. Even though joint angular motion profile is similar between the two conditions, statistical analysis of differences in the joint angle data showed significant differences with respect to the magnitude of a few kinematic features (Table 1). The average hindlimb joint angle curves indicates the stance (0–60%) and swing (60–100%) phases of the gait cycle.

**Table 1:** Joint angle data.

	**Hip (degrees)**		**Knee (degrees)**		**Ankle (degrees)**		**MTP (degrees)**	
	**2D**	**3D**	**2D**	**3D**	**2D**	**3D**	**2D**	**3D**
IC	97 ± 2.7	95 ± 11.4	140 ± 5.6	133 ± 6.1	145 ± 4.9	138 ± 6.1^§^	175 ± 3.8	171 ± 5.5
STANCEmin	97 ± 2.7	95 ± 11.4	106 ± 2.7	104 ± 8.8	129 ± 3.9	131 ± 6.2	164 ± 9.0	164 ± 4.4
STANCEmax	118 ± 4.5	111 ± 13.6	140 ± 5.5	133 ± 6.0	153 ± 5.3	150 ± 7.2	179 ± 0.7	178 ± 2.8
TO	117 ± 4.7	111 ± 13.5	110 ± 2.5	105 ± 6.7	152 ± 4.7	151 ± 6.8	167 ± 4.7	174 ± 4.6
SWINGmin	95 ± 3.4	95 ± 11.0	86 ± 9.7	94 ± 9.1	115 ± 6.8	125 ± 4.8^§^	144 ± 1.7	156 ± 7.4^*^
SWINGmax	117 ± 4.7	112 ± 13.5	135 ± 5.8	132 ± 6.2	153 ± 4.6	153 ± 5.7	174 ± 2.8	179 ± 0.3^**^

*IC, initial contact; STANCEmin, minimal angle during stance; STANCEmax, maximal angle during stance; TO, toe-off; SWINGmin, minimal angle during swing; SWINGmax, maximal angle during swing. Significantly different from 2D;*

* *P < 0.05;*

** *P < 0.01*.

§ *Different from 2D with P < 0.05 in the initial pairwise t-test but not confirmed at this significance level by the bootstrapping procedure (see **Figure 5**)*.

**Figure 4:**
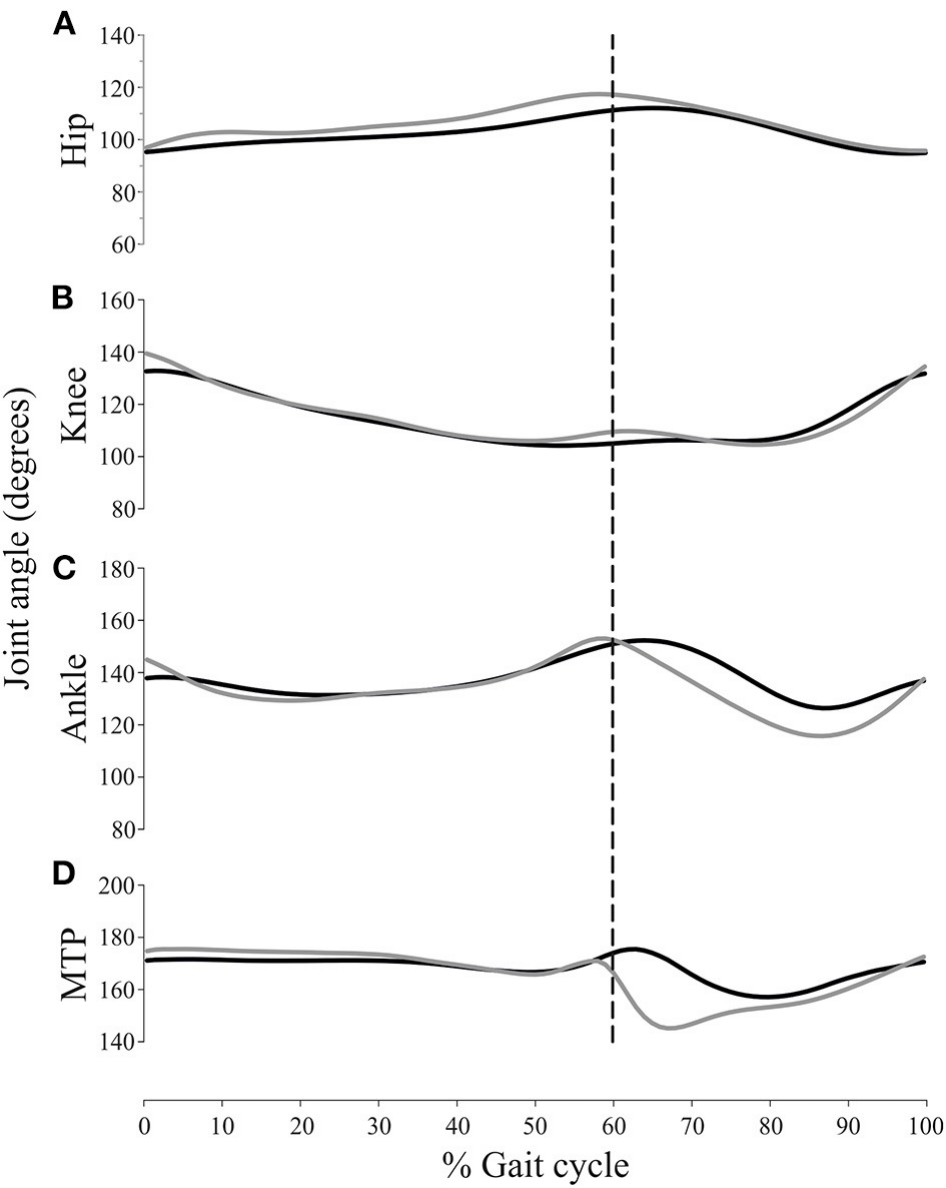
Mean values for the joint angular positions of the hip **(A)**, knee **(B)**, ankle **(C)** and metatarsophalangeal joint (MTP) **(D)**, using the 2D (gray trace) and 3D (black trace) approach. Stance and swing phases were normalized. The stance duration was set at 60% of the gait cycle duration. The vertical dashed line corresponds to the stance–swing transition.

Joint angle 2D and 3D measurements of the MTP joint were significantly different during the swing phase (Table 1). Likewise, the minimal ankle joint angle during the swing phase and its angle during IC also changed when measured by 2D or 3D methods, whereas the hip and knee joint angles did not change with the 2D or 3D setups. The hip and knee flexion/extension angles were most consistent between the two methods, i.e. the smallest relative effect was realized by out-of-plane motion. However, ankle and metatarsophalangeal joint flexion was higher throughout the swing phase of the gait cycle. As can be seen in Figure 4, a subtle but consistent increase in flexion was noted for the 3D hip and metatarsophalangeal joint assessment during the stance phase.

**Figure 5:**
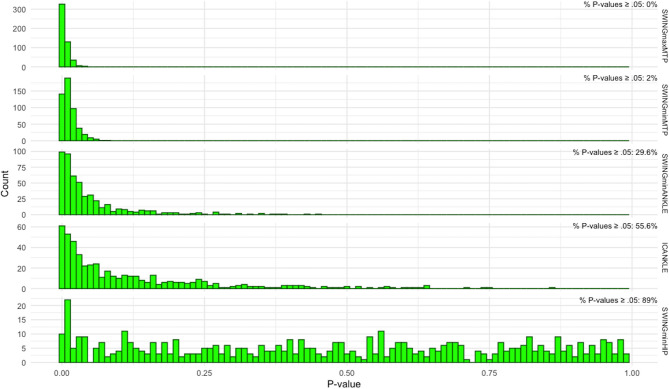
The *P*-values distribution obtained from 500 pairwise *t*-tests using bootstrapped samples of the original 2D and 3D data. The top four histograms are from metatarsophalangeal and ankle angle with significantly different amplitudes during sheep gait when measured by 2D or 3D methods as found initially by Student's pairwise *t*-test. In these histograms, the distribution is greatly shifted leftwards, since a large proportion of bootstrapped *t*-tests had very small *P*-values. Displayed on the top-right of each histogram is the percentage of bootstrapped *t*-tests with *P*-value ≥ 0.05. For the two metatarsophalangeal angles (top two histograms), the percentage does not reach 5%, signifying that more than 95% of the *t*-tests had a *P*-value below the 0.05 significance level, thus in accordance with the strict definition of statistical significance. For the ankle angles, the number of *t*-tests with *P*-values above the significance level surpassed 5%, although most of the *P*-values were less than 0.05 (please note the different *y*-scale for each histogram). The histogram in the bottom graph displays the *P*-values distribution for one of the hip kinematic variables. Here, the *P*-values are distributed uniformly across the probability interval 0 to 1, compatible with the distribution under the null hypothesis. SWINGmaxMTP, maximum amplitude of the metatarsophalangeal joint during the swing phase; SWINGminMTP, minimum amplitude of the metatarsophalangeal joint during the swing phase; SWINGminANKLE, minimum amplitude of the ankle joint during the swing phase; ICANKLE, amplitude of the ankle angle at the instant of initial contact; SWINGminHIP, minimum amplitude of the hip joint during the swing phase.

In the two metacarpophalangeal angles measured differently in 2D or 3D, the percentage of *t*-tests with *p*-values equal or above 0.05 was <5% (Figure 5), so we may confidently reject the null hypothesis and confirm that for the metacarpophalangeal there are significant differences in the maximum and minimum angle amplitudes during the swing phase of the sheep gait when measured in 2D or 3D. For the other two parameters, the minimum ankle angle during the swing phase and the ankle angle amplitude at the initial contact of stance, the evidence is less strong. Although the *p*-values for the two ankle angles are not uniformly distributed, as expected under the null hypothesis (Figure 5), the percentage of *p*-values that were above the significance level was beyond 5%. Therefore, for the ankle parameters we may not decidedly claim the 2D and 3D methods produce different outcomes.

Intra-trial, inter-step ICC values for joint angles measured either in 2D or 3D are presented in Table 2. ICC values for angle measurements were all above 0.75 with a large majority being well above 0.90.

**Table 2:** Intra-class correlation values for inter-step joint angle data.

	**Hip (degrees)**		**Knee (degrees)**		**Ankle (degrees)**		**MTP (degrees)**	
	**2D**	**3D**	**2D**	**3D**	**2D**	**3D**	**2D**	**3D**
IC	0.978	0.999	0.987	0.975	0.986	0.950	0.969	0.989
STANCEmin	0.979	0.999	0.989	0.998	0.985	0.994	0.996	0.996
STANCEmax	0.994	0.999	0.987	0,979	0.992	0.992	0.893	0.978
TO	0.994	0.999	0.994	0.999	0.986	0.991	0.972	0.984
SWINGmin	0.988	0.999	0.989	0.998	0.994	0.986	0.779	0.991
SWINGmax	0.994	0.999	0.977	0.977	0.985	0.991	0.927	0.748

*IC, initial contact; STANCEmin, minimal angle during stance; STANCEmax, maximal angle during stance; TO, toe-off; SWINGmin, minimal angle during swing; SWINGmax, maximal angle during swing*.

## Discussion

Despite their relevance for medical research, little is known about the locomotion in the ovine model, and next to nothing about the 3D kinematics of the hindlimb. As previously mentioned, this study was the first to compare joint hindlimb kinematics in clinically healthy sheep during treadmill walking calculated by a 2D and a 3D method. In the present study, to adequately evaluate the 2D and 3D interventions, the kinematic parameters were collected from the same trials (22).

The results of the reported 2D and 3D kinematic data revealed that although similar joint angular waveforms, statistically significant differences were detected at selected events of the gait cycle. The present study highlights significant differences for the metatarsophalangeal joint, and possibly also for the ankle joint, whereas the magnitude of hip and knee joints were nearly identical for both conditions. This finding could be the result of transverse plane motion of the most distal joints, which could not be accounted for in the 2D video assessment (23). Our 3D sagittal model provides accurate data and is not subject to the typical errors (such as parallax and perspective) that occur when the researchers are collecting with a 2D methodology. It should be noted that, failure to analyze both transverse and frontal plane kinematics data limits the understanding of the joint motion in 3D. However, sagittal plane kinematics has been the focus of motion analysis in the neuroscience field. Mostly because there is a quantitatively large degree of motion in the sagittal plane compared to the transverse and frontal planes.

It is reasonable to assume that different descriptions of the kinematic data collection methodology and distinct ovine breeds used in previously published studies, have a drastic influence on the results among laboratories. Segment and joint kinematics calculations are influenced by marker misplacement and has been considered to have a significant impact on joint angles being investigated (24). In order to minimize these errors, one experienced researcher was responsible for positioning the markers over palpable bony landmarks. Skin movement relative to the underlying bone is of critical relevance for animal gait research (25, 26).

In addition, due to their quadrupedal nature, skin motion artifacts at the knee joint is the most relevant source of error when estimating hindlimb joint kinematics, due to a more extensive skin attachment from the proximal hindlimb to the lateral torso when compared to humans (27).

Our data on 3D knee joint excursion was made possible by using radiography and applying the anatomical findings to biomechanical principles. The knee joint should operate aligned with the plane defined by the greater trochanter, the knee and the lateral malleolus. We may consider the knee as a hinge joint with 1 degree of freedom, moving from extension into full flexion along the sagittal plane (28). Fisher et al. (29) used for the first time in dogs a biplanar, high-frequency fluoroscopy and a 3D optoelectric system. Despite the fact that their description of the canine hindlimb kinematics is much more accurate, however, the cost of equipment, the time-consuming data processing and analysis, and the threat of prolonged radiation exposure make these options unrealistic for the most laboratories involved in spinal cord and peripheral nerve regeneration studies (29).

In order to make a pertinent comparison with our investigation, we only selected the studies where the picture of 3D trajectories painted by the hindlimb joints was made during ovine treadmill walking. Tapper et al. in 2004 were pioneers in describing the dynamic *in vivo* 3D kinematics of the ovine stifle joint (30). The flexion-extension range for knee joint along the sagittal plane almost resembles ours. Tapper's kinematic data on 5 adult female Suffolk-cross sheep (body weight = 77 ± 8 kg) were recorded at 120 Hz using a 4-camera 3D motion capture system, while the animals walked on a treadmill with a velocity of 0.89 m/s. However, the method presented by these authors is invasive and may alter joint kinematics. Valentin et al. captured data on 7 mature female Austrian Mountain sheep (average weight = 72 ± 7 kg) at 120 Hz using a 10-camera 3D motion capture system, while the sheep walked (1.11–1.16 m/s) on a treadmill (5 treadmill sessions). Their kinematic study did not include hindlimb joints excursion, they only assessed movement cycle duration, vertical trunk movement, stride height, stride length and percentage of movement cycle at stance (31). Safayi et al. quantified interlimb coordination and the kinematics of the ankle joint. They used 18 mature (4 male and 14 female) Polypay and Suffolk sheep (average weight = 70) that were recorded at 100 Hz using a 6-camera 3D motion capture system, while the animals moved on a treadmill with a velocity of 1.04 m/s (3 treadmill sessions). As in our study, the ankle joint flexion-extension range during stance and swing was about 36 ± 6 (32). Two years later, the same research group provided an elegant description of the 3D kinematics of the ankle joint from 6 sheep pre- and post-spinal cord injury, using the protocol described above (7). Very recently, a ground-breaking description of the ovine knee joint before and after open surgical procedure (arthrotomy) was reported (33). These authors captured data on 20 mature female Suffolk cross sheep (average weight = 75 ± 5 kg) at 120 Hz and 400 Hz using a 3D motion capture system. In total, a minimum of 100 non-consecutive strides were analyzed with a treadmill velocity of 0.9 m/s. From this investigation, it can be concluded that traumatic knee injury resulted in reduced joint angular velocity.

There are several advantages of using a treadmill, including ambulation within a small area, accurate control of speed and gradient and the ability to capture repeated gait cycles (34). During treadmill walking, animals can walk with a more stable gait because of the constant treadmill walking speed, improving intra-session and inter-session reliability of measurements (35). Even with domestication, sheep retain their gregarious instinct, the desire to stay together for protection (36). In order to reduce the negative impact of being separated from the rest of the flock, the same handler trained all sheep daily to walk on the treadmill 2 weeks before the kinematics testing rewarding them with food. This resulted in a substantial reduction in the amount of time necessary for an accurate data acquisition and in fewer animals being used. To improve the data collection sheep, require initial familiarization to walk on a moving belt to attenuate some treadmill-related differences. Different acclimatization protocols to treadmill walking have been described in the literature, but most of the studies suggest over 6–10 training sessions for the measurement of biomechanical variables (29, 30). In the present study the speed of the treadmill was set to 1.2 m/s, this velocity is now widely accepted as walking locomotion for sheep (37).

We found a high degree of stability in sheep's hindlimb motion during treadmill motion, as a result of an excellent inter-step reliability observed for all angle measurements. Such very high reliability scores are in accordance with the highly controlled conditions during data acquisition, in particular the sheep's steady gait velocity imposed by treadmill walking.

The ICC values described in this study, allows to accurately measure hindlimb angular joints, recorded with a 2D or 3D set-up, and predict the recovery of function in experimental neurological research.

In summary, this research highlights the inadequacy of a 2D kinematic testing in clinically normal sheep during treadmill walking, mostly during swing phase. Moreover, it is expected that in several neurological disorders the pathological gait of sheep may contain substantial 3D movement components from both phases of the gait cycle. The present study established fundamental 3D kinematic characteristics of the sagittal motion that take place at each joint of the hindlimb in the ovine model during treadmill walking. The kinematic results presented here should provide direction for future gait analysis studies that evaluate the hindlimb joint behavior in the sheep model. In the future, this experimental model may serve as an effective tool to evaluate and compare pathological gait patterns and improve our knowledge on kinematic features associated with different neurological conditions.

## Data Availability Statement

The original contributions generated for the study are included in the article/Supplementary Material, further inquiries can be directed to the corresponding author/s.

## Ethics Statement

This investigation was approved by the Institutional Animal Care and Use Committee of the University of Trás-os-Montes e Alto Douro (IACUC Approval No. 6/2015). All procedures were performed with the approval of the Portuguese Veterinary Authorities, in accordance with the EU Directive 2010/63/EU for animal experiments.

## Author Contributions

CD, AM, and AV conceived and designed the study. CD, JC, BF, LM, JP, VF, and PC helped collected the data. VF, PC, and PA-d-S contributed to define the kinematic protocol and data analysis. PA-d-S performed the statistical analysis. SR, AM, and AV contributed to analysis and interpretation of data and to manuscript supervision. All authors contributed to manuscript revision, read and approved the submitted version.

## Conflict of Interest

The authors declare that the research was conducted in the absence of any commercial or financial relationships that could be construed as a potential conflict of interest.

## Publisher's Note

All claims expressed in this article are solely those of the authors and do not necessarily represent those of their affiliated organizations, or those of the publisher, the editors and the reviewers. Any product that may be evaluated in this article, or claim that may be made by its manufacturer, is not guaranteed or endorsed by the publisher.

## Supplementary Material

Video S1 During training procedure sheep were allowed to become familiarized with the treadmill and given food rewards as positive reinforcement.

Video S2 Sheep on treadmill with reflective motion capture markers in place.
